# Effect of Small Polyanions on In Vitro Assembly of Selected Members of Alpha-, Beta- and Gammaretroviruses

**DOI:** 10.3390/v13010129

**Published:** 2021-01-18

**Authors:** Alžběta Dostálková, Barbora Vokatá, Filip Kaufman, Pavel Ulbrich, Tomáš Ruml, Michaela Rumlová

**Affiliations:** 1 Department of Biotechnology, University of Chemistry and Technology, 166 28 Prague, Czech Republic; dostalkl@vscht.cz (A.D.); kaufmanf@vscht.cz (F.K.); 2 Department of Biochemistry and Microbiology, University of Chemistry and Technology, 166 28 Prague, Czech Republic; vokataa@vscht.cz (B.V.); ulbrichp@vscht.cz (P.U.); rumlt@vscht.cz (T.R.)

**Keywords:** IP6, polyanion, RSV, MLV, M-PMV, immature, hexamer, assembly, SP domain, CAH

## Abstract

The assembly of a hexameric lattice of retroviral immature particles requires the involvement of cell factors such as proteins and small molecules. A small, negatively charged polyanionic molecule, myo-inositol hexaphosphate (IP6), was identified to stimulate the assembly of immature particles of HIV-1 and other lentiviruses. Interestingly, cryo-electron tomography analysis of the immature particles of two lentiviruses, HIV-1 and equine infectious anemia virus (EIAV), revealed that the IP6 binding site is similar. Based on this amino acid conservation of the IP6 interacting site, it is presumed that the assembly of immature particles of all lentiviruses is stimulated by IP6. Although this specific region for IP6 binding may be unique for lentiviruses, it is plausible that other retroviral species also recruit some small polyanion to facilitate the assembly of their immature particles. To study whether the assembly of retroviruses other than lentiviruses can be stimulated by polyanionic molecules, we measured the effect of various polyanions on the assembly of immature virus-like particles of Rous sarcoma virus (RSV), a member of alpharetroviruses, Mason-Pfizer monkey virus (M-PMV) representative of betaretroviruses, and murine leukemia virus (MLV), a member of gammaretroviruses. RSV, M-PMV and MLV immature virus-like particles were assembled in vitro from truncated Gag molecules and the effect of selected polyanions, myo-inostol hexaphosphate, myo-inositol, glucose-1,6-bisphosphate, myo-inositol hexasulphate, and mellitic acid, on the particles assembly was quantified. Our results suggest that the assembly of immature particles of RSV and MLV was indeed stimulated by the presence of myo-inostol hexaphosphate and myo-inositol, respectively. In contrast, no effect on the assembly of M-PMV as a betaretrovirus member was observed.

## 1. Introduction

Retroviruses assemble immature particles either in the cytosol or at the plasma membrane of the host cell. Betaretroviruses, including Mason-Pfizer monkey virus (M-PMV) and mouse mammary tumor virus (MMTV), are representatives of the morphological type D, which assembles intracytoplasmic A-type particles (ICAPs) in the pericentriolar region. ICAPs are then transported to the plasma membrane where budding and maturation occur. The assembly of retroviruses belonging to the morphological group C is initiated by the interaction of a few molecules of Gag polyprotein with the dimer of viral genomic RNA in the cytosol [1,2]. This complex is then transported to the plasma membrane, where the Gag molecules multimerize to form immature particles. The processes of budding and maturation are similar for immature particles of both D type and C type.

Immature particles of all retroviruses are composed of radially arranged Gag polyprotein precursors, which assemble into a hexagonal lattice [3,4,5,6]. Gag contains three major domains common to all retroviruses: matrix (MA), capsid (CA), and nucleocapsid (NC). Although the amino acid sequences of these domains vary across the retroviral genera, their secondary structures and functions remain preserved. The MA domain is responsible for directing the Gag polyproteins to the assembly site [7,8,9,10,11,12,13,14,15]. Multimerization of Gag is then mediated by the highly structurally conserved CA domain (for a review, see [16]). The CA domain consists of two globular independently folded N-terminal (NTD) and C-terminal (CTD) subdomains connected by a flexible linker. Retroviral NTDs are comprised of six or seven α-helices and one β-hairpin and define the morphology of immature particles [3,4,5,6]. Detailed cryo-electron microscopy (cryo-EM) structural analysis of HIV-1, M-PMV, RSV, and MLV revealed different arrangements of NTDs in the immature particles of these retroviruses [3,4,5,6], whereas the arrangement of CTD is more conserved. The inter- and intramolecular interactions of the CTD′s four α-helices are responsible for the formation and stabilization of the Gag hexameric lattice. The lattice is connected by interhexameric interactions facilitated by CTD–CTD and intrahexameric interfaces mediated by both CTD–CTD and NTD–NTD [3,4,5,6]. Downstream of the CA domain is the NC domain, which is responsible for the recognition and specific interaction of Gag with viral genomic RNA.

A region between the HIV-1 CA and NC domains, spacer peptide 1 (SP1), consists of 14 amino acid residues and was identified to be critical for immature particle formation [17]. Circular dichroism analysis of the region spanning the last eight amino acids of the HIV-1 CA CTD and two-thirds of SP1 showed a concentration-dependent shift corresponding to a transition from unstructured peptide to amphipathic α-helix during the oligomerization of Gag [18]. In the immature hexameric lattice, six α-helices of these regions form a rod-like structure, called a six-helix bundle. This structure contributes to the formation of the immature particle and its stability [19,20,21]. In addition to lentiviruses, alpharetroviruses also contain a spacer peptide (SP) linker between the CA and NC domains. Mutational analysis of RSV showed that apart from the 12-amino-acid-residue-long SP, its adjacent upstream and downstream sequences, i.e., the last eight amino acids of the CA CTD and the first four residues of the NC domain, are also critical for the assembly of RSV immature particles [22,23]. Cryo-electron tomography of RSV immature particles revealed that below the CA domain, a hollow cylinder of the density, corresponding to the six-helix bundle observed for SP1 of HIV-1, is also present in RSV [6,16,23]. Moreover, a six-helix bundle model generated for RSV SP [23] fit into the density [6].

Even though the NC domains of both gamma- and betaretroviruses directly follow the CA domain without any linker, the last few amino acids of the CA CTD and the first few amino acids of the NC domain were shown to be critical for the assembly of immature particles [24,25,26,27]. The C-terminus of the MLV CA CTD region containing 42 mostly charged amino acids forms a charged α-helix (CAH), which is essential for the assembly of immature particles [24,27]. The cryo-electron tomographic structure of MLV immature particles revealed that the N-terminal 15 residues of the CAH (P222-K236) form the six-helix bundle [4]. A mutational study showed that the CAH participated during the formation of the immature particles in *Escherichia coli* [24]. In contrast to gammaretroviruses, the cryo-EM structure of immature M-PMV particles showed a lack of any helical motif connecting the CA and NC domains. Nevertheless, the 33-amino-acid region between the M-PMV CA and NC was suggested to functionally mimic the SP domain [25,26]. The sequence was named SP-like domain-derived peptide (SPLP) and was shown to play an indispensable role during the assembly of immature viral particles both in vivo and in vitro [26]. In addition, the C-terminus of the CA in M-PMV encodes a sequence of three basic amino acid residues (R201-K203), the so-called RKK motif [28]. Two neighboring CA molecules form a basic patch on the underside of the CA layer in immature Gag arrays, which was shown to be essential for the assembly and RNA incorporation [29].

The assembly of immature particles also requires various cellular factors facilitating several steps of this process. Among the examples of the cellular proteins that bind RNA and Gag are, e.g., Staufen I, which facilitates RNA encapsidation into the immature particle [30], Golgi-localized γ-ear containing Arf-binding (GGA) proteins, participating during trafficking of the preassembly complex to the plasma membrane [31], or ATP-binding cassette protein E1 (ABCEI), which is involved in Gag multimerization [32]. Apart from proteins, the cells comprise numerous low-molecular-weight factors that are involved in the formation and stabilization of immature retroviral particles. Recently, the region between the CA CTD and NC domain of HIV-1 was identified as a binding site for myo-inositol hexaphosphate (IP6), which was shown to be an essential co-factor for HIV-1 assembly [33]. IP6 interacts with the two lysine residues K290 and K359, located in a major homology region (MHR) in CA CTD and at the top of the six-helix bundle, respectively. IP6 was determined to be crucial for the stability of six-helix bundle and Gag hexamer formation [33,34]. Recently, Dick et al. showed that IP6 stimulates the assembly of other lentiviral members and, based on the sequence homology of MHR and CA-SP regions, proposed that all lentiviruses bind IP6 by similar binding pockets [35].

Considering that the CA–NC junctions of different retroviral genera play a similar structural–functional role during immature particle formation, it is plausible that this region might be a common binding site for IP6 or other co-factor(s) stimulating proper stability and Gag hexamer formation. Here, we determined the effect of several polyanions, including IP6, on stimulating the in vitro assembly of the members of three retroviral genera: RSV (alpharetroviruses), M-PMV (betaretroviruses) and MLV (gammaretroviruses). The polyanions used in our study, namely myo-inositol hexaphosphate (IP6), myo-inositol (myo-In), glucose-1,6-bisphosphate (G-2P), myo-inositol hexasulphate (IS6) and mellitic acid (MeA), were selected according to their size and charge distribution. To test and quantify the effect of these molecules on the assembly, we combined the principle of the in vitro assay Fast Assembly Inhibitor Test for HIV-1 (FAITH) [36,37] with the protocols established for in vitro assembly of particular retroviral Gag-derived proteins—RSV 25p10CASPNC [38], MLV ∆10CANC [39] and M-PMV ∆ProCANC [40,41]. The data showed that polyanions affected the assembly of retroviruses with the six-helix bundle at the CA and NC junction and forming immature particles at the plasma membrane. In contrast, the polyanions did not show any effect on assembly efficiency of M-PMV lacking the six-helix bundle structure and assembling within the cytoplasm. Whether these differences might be connected to virus morphogenesis remains unclear.

## 2. Materials and Methods

### 2.1. Expression Vector

M-PMV ∆ProCANC and MLV ∆10CANC expression vectors were prepared as described earlier [39,41]. Plasmid for RSV protein expression, pTriExT-Smt3-25p10-CA-NC RSV, was provided by Dr. Tibor Fuzik (CEITEC, Brno, Czech Republic).

### 2.2. Protein Expression and Purification

RSV 25p10CASPNC and M-PMV ∆ProCANC proteins were purified as described previously [38,41,42,43]. MLV ∆10CANC and its derived mutant proteins were purified as described previously [24]. The purified proteins were analyzed by SDS-PAGE and verified by Western blot analysis.

### 2.3. Quantification of Assembly Efficiency of RSV 25p10CASPNC

RSV immature virus-like particles were formed from purified bacterially expressed proteins and nucleic acid by overnight dialysis. Briefly, 6 µg of RSV 25p10CASPNC protein (1.35 μM) was mixed with 0.6 µg of dual-labeled oligonucleotide (tqON) carrying black hole quencher 1 (BHQ) and fluorescent dye 6-carboxyfluorescein (FAM) and polyanions at concentrations ranging from 0.06 to 0.45 µM in RSV assembly buffer containing 50 mM Tris pH 8.0, 0.5 M NaCl, 1 µM ZnSO_4_ and 1 mM tris(2-carboxyethyl)phosphine (TCEP). The mixture was dialyzed against a dialysis buffer containing 50 mM Tris pH 8.0, 1 µM ZnCl_2_ and 100 mM NaCl overnight at 4 °C. The mixture of assembled particles was then transferred to a 96-well plate and Exonuclease I (ExoI) together with Mg^2+^ ions was added. The fluorescence released after cleavage of tqON by ExoI was measured using the spectrophotometer Tecan M200Pro, and the assembly efficiency was calculated at 40 min as described previously [36,37]. To calculate assembly efficiency, the formula E = 100 × ∆F_2_/∆F_1_ was used, in which ∆F1 corresponds to the difference between the relative fluorescence of tqON and the relative fluorescence of tqON in the presence of 25p10CASPNC, and ∆F2 corresponds to the difference between the relative fluorescence of tqON and the relative fluorescence of tqON in the presence of 25p10CASPNC and polyanions.

### 2.4. Quantification of Assembly Efficiency of MLV ∆10CANC

Similar to the case of RSV, MLV ∆10CANC protein and mutant proteins R225A and R225A/R228A/R230A were assembled by overnight dialysis at 4 °C. A mixture of 60 µg of MLV ∆10CANC protein (13.5 µM), 6 µg of tqON and polyanions at a concentration ranging from 0.6 to 4.5 µM in MLV assembly buffer (20 mM Tris pH 8.0, 500 mM NaCl, 50 µM ZnCl_2_, 1 mM phenylmethylsulfonyl fluoride (PMSF) and 10 mM dithiothreitol (DTT) was dialyzed against the dialysis buffer. Then, the reaction mixture was transferred to a 96-well plate, ExoI together with Mg^2+^ ions was added, the fluorescence was measured and the assembly efficiency was calculated at 50 min, as was described for RSV.

### 2.5. Quantification of Assembly Efficiency of M-PMV ∆ProCANC

First, 60 µg of M-PMV ∆ProCANC protein (14.3 µM) was mixed with 6 µg of tqON and polyanions at a concentration from 0.6 to 5 µM in M-PMV assembly buffer, which consists of 50 mM phosphate, 0.5 M NaCl, 0.05% β-ME and 1 µM ZnSO_4_, pH 7.5. The mixture was dialyzed against the dialysis buffer overnight at 4 °C. The fluorescence was measured in a 96-well plate after adding ExoI together with Mg^2+^ ions. Assembly efficiency was then calculated at 40 min, as was described for RSV.

### 2.6. Transmission Electron Microscopy

The in vitro assembled particles were visualized by transmission electron microscopy of negatively stained samples. The particles were deposited on a carbon-coated copper grid for 2–5 min. The grid with the sample was dried, washed twice with deionized water and negatively stained with 4% sodium silicotungstate (pH 7.4) or 1% uranyl acetate for 30 s. The samples were visualized using a JEOL JEM-1010 transmission electron microscope (JEOL, Tokyo, Japan) at 80 kV. Images were recorded with an AnalySIS MegaView III CCD camera.

## 3. Results

### 3.1. Preparation of Gag-Derived Proteins

**Figure 1:**
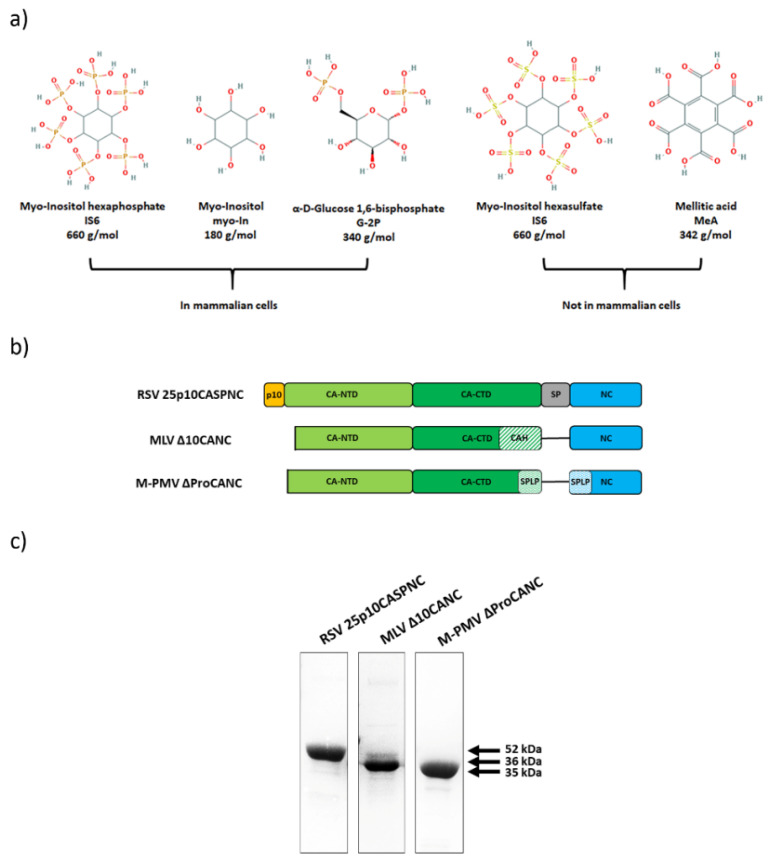
Gag-derived CANC proteins of Rous sarcoma virus (RSV), murine leukemia virus (MLV) and Mason-Pfizer monkey virus (M-PMV) used in this study. (**a**) Chemical structures of the used polyanions: myo-inostol hexaphosphate (IP6), myo-inositol (myo-In), glucose-1,6-bisphosphate (G-2P), myo-inositol hexasulphate (IS6) and mellitic acid (MeA). (**b**) Schematic representation of CANC proteins of RSV, MLV and M-PMV. (**c**) Coomassie blue-stained SDS-PAGE gel, documenting the purity of the proteins.

**Figure 2:**
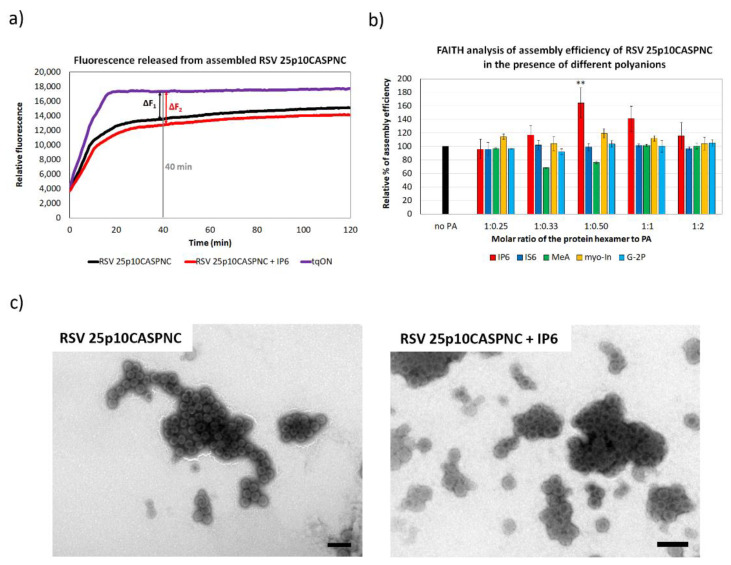
The efficiency of RSV 25p10CASPNC protein assembly in the presence of polyanions. (**a**) Fluorescence emission curve demonstrating the kinetics of degradation of free dual-labeled oligonucleotide (tqON) in the absence (violet curve) or presence (black curve) of RSV 25p10CASPNC: ∆F1 represents the difference between the relative fluorescence of tqON and tqON in the presence of 25p10CASPNC, and ∆F2 corresponds to the difference between the relative fluorescence of tqON and tqON in the presence of 25p10CASPNC and polyanions. (**b**) Percentage of assembly efficiency of RSV 25p10CASPNC protein in the presence of different concentrations of IP6 (red), IS6 (royal blue), MeA (green), myo-In (yellow) and G-2P (azure blue) at indicated ratios of the protein hexamer to IP6. *p*-values were assessed by ANOVA using the Tukey–Kramer test (**, *p* < 0.01). (**c**) TEM images of negatively stained RSV 25p10CASPNC protein assembled in the presence of tqON in the absence or presence of IP6 at final concentration 1.1 µM (the ratio of the protein hexamer to IP6 was 1:0.5). Bars represent 200 nm.

Assembly of retroviral particles is a critical step of the viral life cycle. It is orchestrated primarily by the capsid and nucleocapsid domains of the Gag polyprotein precursor; however, various cell factors such as proteins or small molecules facilitate this process. To study the effect of selected small molecules (Figure 1a) on the assembly of RSV, MLV and M-PMV, we produced the Gag-derived CANC fusion proteins of these retroviruses in *E. coli* BL21(DE3), purified them and analyzed them by SDS-PAGE (Figure 1b,c). These proteins were then assembled in vitro into immature virus-like particles using the previously described protocol adapted for each retrovirus [38,39,41,44]. To determine the efficiency of assembly, we utilized the principle of the previously developed in vitro fluorescence assay FAITH [36]. To trigger the assembly of the CANC protein, this assay uses a dual-labeled oligonucleotide (tqON) carrying both quencher BHQ and fluorescent dye FAM. Following the assembly, when tqON is bound to the NC domain and becomes hidden inside the assembled particles, Exonuclease I (ExoI) is added. ExoI-mediated degradation of free tqON leads to an increase in fluorescence due to the separation of fluorophore from its quencher (Figure 2a). The efficiency of assembly is then calculated based on released fluorescence measured by a spectrophotometer. None of the tested polyanions affected the activity of ExoI as was previously shown [37]. To calculate the assembly efficiency, the formula E = 100 × ∆F2/∆F1 was used, in which ∆F1 corresponds to the difference between the relative fluorescence of tqON and the relative fluorescence of tqON in the presence of retroviral Gag-derived protein, and ∆F2 corresponds to the difference between the relative fluorescence of tqON and the relative fluorescence of tqON in the presence of retroviral Gag-derived protein and polyanions (Figure 2a).

### 3.2. The Effect of Polyanions on the Assembly of RSV Immature Virus-Like Particles

Similar to HIV-1, the RSV CA-CTD-SP region also adopts a helical conformation in immature particles [6]. In the HIV-1 hexameric lattice, this helical segment forms a six-helix bundle [3,19,45,46], which was also observed in RSV virus-like particles [6]. Based on this structural similarity, the RSV CA-CTD-SP region can represent a potential binding site for a small molecule that would enhance the assembly and stability of the immature particles. To analyze this possibility, RSV immature virus-like particles were assembled in vitro in the presence of various polyanions, including myo-inostol hexaphosphate (IP6), myo-inositol (myo-In), glucose-1,6-bisphosphate (G-2P), myo-inositol hexasulphate (IS6) and mellitic acid (MeA), and their impact on the assembly was analyzed by FAITH. RSV immature particles were assembled during overnight dialysis of RSV 25p10CASPNC protein in the presence of tqON in a range of concentrations of selected polyanions in RSV assembly buffer. The assembly efficiency was measured as an increase in fluorescence after the addition of ExoI and calculated from the formula E = 100 × ∆F_2_/∆F_1_, as was described above (Figure 2a). The only significant increment in RSV 25p10CASPNC assembly efficacy was observed for IP6. At the concentration ratio of protein hexamer to IP6 1:0.5, IP6 increased the assembly efficiency by 1.6-fold. In contrast, the other polyanions did not affect the assembly (Figure 2b). The shape and size of the particles assembled in the presence of IP6 were verified by TEM (Figure 2c).

### 3.3. The Effect of Polyanions on the Assembly of MLV Immature Virus-Like Particles

**Figure 3:**
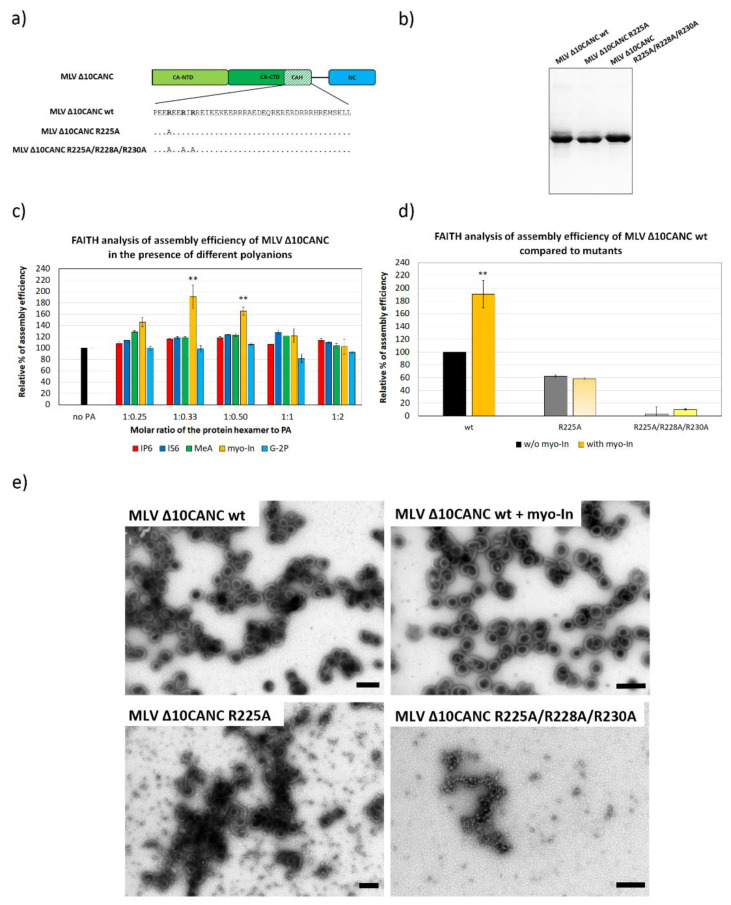
The efficiency of MLV ∆10CANCwild type (wt) and mutant proteins’ assembly in the presence of selected polyanions. (**a**) Schematic representation and amino acid sequence of MLV ∆10CANCwt and R225A and R225A/R228A/R230A mutants. (**b**) Coomassie blue-stained SDS-PAGE gel, documenting the purity of MLV ∆CANC wt and R225A and R225A/R228A/R230A mutants. (**c**) Percentage of assembly efficiency of MLV ∆10CANC wt protein in the presence of different concentrations of IP6 (red), IS6 (royal blue), MeA (green), myo-In (yellow) and G-2P (azure blue) at indicated ratios of protein hexamer to polyanions. *p*-values were assessed by ANOVA using the Tukey–Kramer test (**, *p* < 0.01). (**d**) Percentage of assembly efficiency of MLV ∆10CANC wt (black column), R225A (dark grey column) and R225A/R228A/R230A (light grey column) compared to the proteins assembled in the presence of 1.2 µM myo-In (yellow). *p*-values were assessed by ANOVA using the Tukey–Kramer test (** *p* < 0.01). (**e**) TEM analysis of negatively stained MLV ∆10CANC wt protein assembled in the absence or presence of myo-In at a final concentration of 1.2 µM (ratio of the protein hexamer to myo-In was 1:0.5) and both the R225A and R225A/R228A/R230A mutants in the absence of myo-In. Bars represent 200 nm.

Although in contrast to HIV-1 and RSV, MLV Gag lacks the cleavable SP domain, the C-terminal forty amino acids of MLV CA form an alpha-helix, which is due to the presence of 32 charged amino acids (16 positively and 16 negatively), also known as a charged assembly helix (CAH). The authors and others have shown previously that the CAH promotes the assembly of immature MLV particles [4,24,27]. Although the resolution of the cryo-electron tomography (cryo-ET) MLV immature particles’ structure for the CAH region was not sufficient for its de novo structure determination, the six-helix bundle model comprising the N-terminal 15 amino acids fitted nicely into the determined electron density [4]. Therefore, it can be presumed that in addition to HIV-1 and RSV, MLV is also capable of adopting the six-helix bundle conformation. Therefore, MLV CAH could represent a potential binding site for polyanions. To analyze this hypothesis, we assembled purified MLV ∆10CANC protein (Figure 3a,b) in the presence of tqON and an increasing amount of the selected polyanions (Figure 3c). The strongest effect on MLV ∆10CANC assembly was repeatedly observed only for myo-In, which increased the assembly efficiency almost twofold (Figure 3c,d). In contrast, other polyanions including IP6, IS6, G-2P and MeA had no significant impact on the assembly of MLV wild type (wt) Gag. Interestingly, the myo-In impact was observed for the protein hexamer:myo-In ratio from 1:0.33, when it culminated, to 1:0.50. However, no effect of myo-In was determined at the ratio of protein hexamer:myo-In 1:1 or higher.

To support our hypothesis that the basic amino acid residues of CAH are responsible for myo-In binding, we reduced the positive charge within the CAH by mutations of the basic residues within the N-terminal 15 amino acids. We selected all basic residues (R225, R228 and R230) at the N-terminus of CAH because only the N-terminal 15 residues of the CAH (P222-K236) were shown to form the six-helix bundle [4]. Two ∆10CANC mutants, carrying single R225A and triple R225A, R228A and R230A mutations (Figure 3a), were purified (Figure 3b) and tested for polyanion binding capacity. The quantitation of the in vitro assembly efficiency of these two mutants, however, showed their impaired ability to assemble compared to the wt (Figure 3d). Interestingly, despite the decreased assembly efficacy, we observed that the R225A mutation did not completely abrogate immature virus-like particles’ formation as shown by using both FAITH and TEM (Figure 3d,e). Although myo-inositol increased the assembly efficiency of the wt, the assembly of R225A was not affected by the presence of myo-In (Figure 3d,e).

### 3.4. The Effect of Polyanions on the Assembly of M-PMV Immature-Like Particles

**Figure 4:**
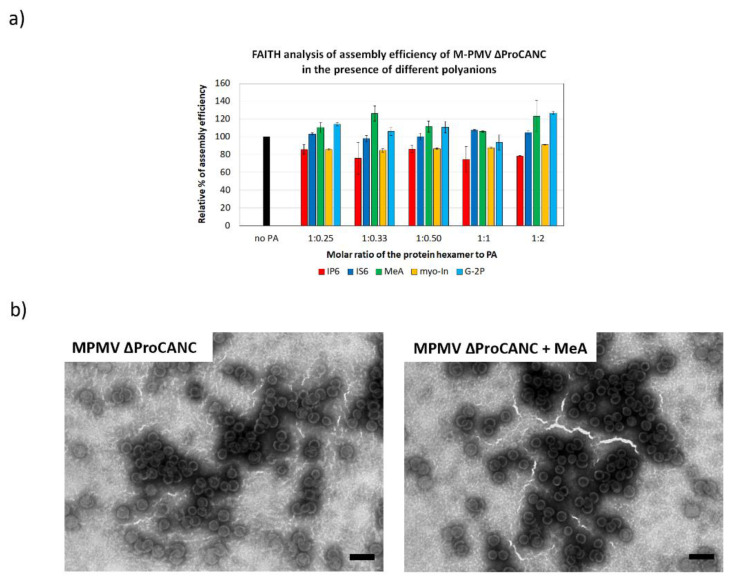
Efficiency of M-PMV ∆ProCANC wt protein assembly in the presence of selected polyanions. (**a**) Percentage of assembly efficiency of M-PMV ∆ProCANC wt protein in the presence of IP6 (red), IS6 (royal blue), MeA (green), myo-In (yellow) and G-2P (azure blue) at indicated protein hexamer to polyanions ratios. (**b**) TEM analysis of negatively stained M-PMV ∆ProCANC wt proteins assembled in the absence (**left**) and presence (**right**) of MeA (the final concentration of MeA was 0.8 µM and the ratio of the protein hexamer to MeA was 1:0.33). Bars represent 200 nm.

Unlike other retroviruses, M-PMV Gag encodes neither the CA-NC spacer peptide nor a charged alpha-helix at the C-terminus of CA CTD [26]. However, the CA CTD of M-PMV contains the RKK motif, which could possibly play a role in polyanion bindings. To analyze whether the formation of M-PMV immature virus-like particles can be stimulated by the addition of polyanion binding, M-PMV ∆ProCANC was assembled in the presence of tqON and increasing concentrations of the selected polyanions. Next, we measured the fluorescence of degraded tqON inversely proportional to the amount of assembled particles. As shown in Figure 4a, the assembly of M-PMV particles was slightly, but statistically insignificantly, increased in the presence of mellitic acid and G-2P (Figure 4a). No positive effect on the assembly was observed for the other polyanions. In contrast, a 20% decrease in the assembly efficiency was observed at all tested concentrations of IP6. Uniform spherical immature virus-like wt M-PMV particles assembled both in the presence and absence of MeA, as confirmed by TEM (Figure 4b).

## 4. Discussion

It was thought that the assembly of retroviral immature particles is stimulated predominantly by protein–protein and protein–nucleic acid interactions of CA–CA and NC–viral genomic RNA, respectively. An increasing amount of evidence, however, shows that interactions of proteins with small molecules also take an essential place in the stimulation of retroviral hexameric lattice formation. The combination of biochemical and structural data provided evidence that one of the small molecules, inositol hexaphosphate (IP6), affects the assembly of HIV-1 immature particles. The involvement of IP6 in the modulation of the retroviral assembly was described twenty years ago [34,47], but the mechanism and structural aspects were discovered only recently [33].

The IP6 molecule consists of a hexagonal carbon ring, with six negatively charged phosphates which bind to two rings of six lysines of MHR and the six-helix bundle and, thus, stabilizes the newly formed hexameric lattice of HIV-1 Gag polyprotein [21,33,34,47]. Recently, another lentivirus, EIAV, has been shown to recruit IP6. Similar to HIV-1, the IP6 molecule within EIAV immature particles binds in the center of the hexamer and is coordinated by two rings of six lysines in the MHR and the six-helix bundle [35]. In addition, in vitro assembly of immature particles of other lentiviruses such as simian immunodeficiency virus (SIV), feline immunodeficiency virus (FIV) and bovine immunodeficiency virus (BIV) has been shown to be stimulated by the presence of IP6 [35].

Although this specific region for IP6 binding may be uniform only among lentiviruses, it seems possible that other retroviral species also recruit some small polyanions to facilitate the assembly of their immature particles. This is supported by the observation that six-helix bundles or similar rod-like structures were observed not only in lentiviral Gag but also in alpharetroviruses such as RSV [6] and gammaretroviruses such as MLV [4]. In this study, we monitored the effect of polyanions on the in vitro assembly of three retroviral genera: RSV (alpharetroviruses), MLV (gammaretroviruses) and M-PMV (betaretroviruses), differing in the region spanning the CA and NC junction. To study the effect of small molecules on the assembly, we selected five polyanionic molecules: myo-inositol hexaphosphate (IP6), myo-inositol (myo-In) and glucose-1,6-bisphosphate (G-2P), which are naturally present in cells, and inositol hexasulphate (IS6) and mellitic acid (MeA), which are synthetic molecules [37].

To mimic the assembly of immature virus-like particles, we used CA–NC derived constructs of RSV, MLV and M-PMV for which in vitro assembly was well established [38,39,40,41,48,49]. The N-terminus of the CA liberated during maturation leads to the formation of a β-hairpin, which is important for the assembly of the mature CA lattice. To achieve immature particle assembly, it is, therefore, necessary to prevent β-hairpin formation by flanking regions upstream of the CA. In this respect, RSV, MLV and M-PMV differ. For proper immature RSV assembly, the C-terminal 25 residues of p10, located upstream of the CA, are essential [38,50]. In contrast, deletions of CA N-termini by one and ten amino acid(s) are required in M-PMV and MLV to prevent the N-terminal β-hairpin formation, respectively [39,41,44].

An alpharetroviruses member, RSV, is the only retrovirus that contains the spacer-peptide region linking the CA and NC domains in addition to lentiviruses. The arrangement of this segment below the CA within the immature particle is similar to that observed for HIV-1, suggesting the presence of a six-helix bundle [6,23]. The lysine residues in RSV are located at different positions compared to lentiviruses; nevertheless, the presence of K412 and K466 close to the MHR and the top of six-helix bundle, respectively, does not rule out the possibility of polyanion binding. Of the tested polyanions, only IP6 affected the assembly of RSV immature particles. However, in contrast to HIV-1 where the addition of IP6 enhances the assembly of HIV-1 particles threefold [37], the efficiency of RSV immature virus-like particles’ assembly in the presence of IP6 was increased by 1.8-fold. Moreover, while the efficiency of immature virus-like HIV-1 particle assembly was increased in a concentration-dependent manner, with a peak at a hexamer:IP ratio of 1:1, the assembly of RSV immature particles was increased significantly at the hexamer-to-IP6 ratio of 1:0.5, and at higher IP6 concentrations, this effect diminished. This ratio corresponds to the presence of IP6 in every second CA hexamer. These data showed that IP6 enhances the assembly of RSV immature particles at the hexamer:IP6 ratio of 1:0.5. Therefore, it can be speculated that the mechanism will differ from that in HIV-1.

While both HIV-1 and RSV have the SP domain as a potential IP6 binding site, the member of the gammaretroviral family, MLV, contains a charged region termed the charge assembly helix (CAH) at the C-terminus of CA. The CAH structural model fit into the cryo-EM electron density map of MLV immature particles, suggesting that this region most likely adopts a structural organization similar to the six-helix bundle [4]. However, the insufficient resolution of this region in the cryo-EM structural analysis did not allow for observing any additional density that would suggest the presence of a cofactor molecule [4]. Our data showed that the in vitro assembly of MLV was not affected by IP6; however, it was significantly (by twofold) increased in the presence of myo-In. Interestingly, this myo-In stimulation was observed only at protein hexamer:myo-In ratios lower than 1:1. The strongest effect was observed at protein hexamer:myo-In ratios of 1:0.33 and 1:0.5, suggesting the presence of this polyanion in each third or second hexamer, respectively. Whether myo-In could bind any of the 16 basic amino acids within CAH remains unclear. To answer this, we mutated arginine residues (R225A and R225A/R228A/R230A) within the CAH N-terminus, which was suggested to adopt the six-helix bundle [4]. Compared to the triple mutant, the single R225A mutant was able to assemble; however, the production of MLV virus-like particles was lower compared to the wild type, which is in agreement with our previous study [24]. In contrast to the MLV ∆10CANC wild-type protein, the addition of myo-In did not stimulate the assembly of the R225A mutant. The conclusions for these data are similar to those that we drew above for RSV: myo-In was found to significantly enhance the assembly of RSV immature particles; however, the mechanism of polyanion binding is unclear.

Although M-PMV Gag lacks both the SP domain and a charged assembly helix, it contains the RKK motif at CA CTD, which was found to be critical for virus particle assembly [29] and the incorporation of viral genomic RNA. Moreover, a detailed mutational analysis of the CA–NC junction clearly showed the importance of 33 amino acid residues between the C-terminus of the M-PMV CA domain and the N-terminus of the NC domain for the assembly of particles. Based on a similar position and function, this M-PMV segment was named as a spacer peptide-like domain [26]. However, this segment does not fold into helical conformation [26]. Our data showed that no polyanions affected the in vitro assembly of M-PMV immature particles.

In summary, our data showed that in contrast to certain structural similarities in the segment connecting the CA–NC regions, only some retroviruses evolved a strategy of using small polyanionic molecules as stabilizers of the hexameric immature lattice. While the assembly of immature particles of HIV-1, and most likely other lentiviruses, is stabilized by IP6 in a concentration-dependent manner, with a peak at the ratio of protein hexamer:IP6 of 1:1, the assembly of the members of alpharetroviruses and gammaretroviruses RSV and MLV, respectively, was found to also be enhanced by polyanions, but the mode of interactions remains unclear.

## Data Availability

Not applicable.
